# Ranking facilitators and barriers of medication adherence by patients with inflammatory arthritis: a maximum difference scaling exercise

**DOI:** 10.1186/s12891-020-03874-2

**Published:** 2021-01-06

**Authors:** M. J. H. Voshaar, J. E. Vriezekolk, A. M. van Dulmen, B. J. F. van den Bemt, M. A. F. J. van de Laar

**Affiliations:** 1 Department Psychology, Health and Technology, University of Twente, Enschede, The Netherlands; 2 Department of Rheumatology, Sint Maartenskliniek, Nijmegen, The Netherlands; 3 Nivel (Netherlands Institute for Health Services Research), Utrecht, The Netherlands; 4 Department of Primary and Community Care, Radboud University Medical Centre, Radboud Institute for Health Sciences, Nijmegen, The Netherlands; 5 Faculty of Health and Social Sciences, University of South- Eastern Norway, Drammen, Norway; 6 Department of Pharmacy, Sint Maartenskliniek, Nijmegen, The Netherlands; 7 Department of Pharmacy, RadboudUMC, Nijmegen, The Netherlands; 8 Department of Clinical Pharmacy and Toxicology, Maastricht University Medical Centre, Maastricht, The Netherlands; 9 Arthritis Centre Twente, Medisch Spectrum Twente & University of Twente, P.O box 50,000, 7500 KA Enschede, The Netherlands

**Keywords:** Inflammatory arthritis, Rheumatoid arthritis, Medication adherence, MaxDiff, Best-worst scaling, DMARDs use

## Abstract

**Introduction:**

Facilitators and barriers of adherence to disease-modifying anti-rheumatic drugs (DMARDs) have been identified by patients with inflammatory arthritis earlier. However, the relative importance from the patients’ perspective of these factors is unknown. Knowledge on this ranking might guide the development of interventions and may facilitate targeted communication on adherence. This study aims to examine 1) the relative importance patients attach to facilitators and barriers for DMARDs adherence, and 2) the relationship between patient characteristics and ranking of these factors.

**Methods:**

One hundred twenty-eight outpatients with inflammatory arthritis; (60% female, mean age 62 years (SD = 12), median disease duration 15 years, IQR (7, 23) participated in a Maximum Difference scaling exercise and ranked 35 items based upon previously identified facilitators and barriers to medication adherence. Hierarchical Bayes estimation was used to compute mean Rescaled Probability Scores (RPS; 0–100) (i.e. relative importance score). Kendall’s coefficient of concordance was used to examine a possible association between patients’ characteristics (i.e. age, sex and educational level) and ranking of the items.

**Results:**

The three most important items ranked by patients were: Reduction of symptoms formulated as “Arthritis medications help to reduce my symptoms” (RPS = 7.30, CI 7.17–7.44), maintaining independence formulated as “I can maintain my independence as much as possible” (RPS = 6.76, CI 6.54–6.97) and Shared decision making formulated as “I can decide –together with my physician- about my arthritis medications” (RPS = 6.48, CI 6.24–6.72). No associations between patient characteristics and ranking of factors were found.

**Conclusions:**

Reducing symptoms, maintaining independency and shared decision making are patients’ most important factors for DMARDs adherence. This knowledge might guide the development of interventions and may facilitate communication between health professionals and their patients on medication adherence.

## Key messages

- *Reducing symptoms, maintaining independency and shared decision making are patients’ most important factors for DMARDs adherence*
- *Facilitators and barriers of DMARDs adherence do not seem to be related to sex, age or education*

## Background

Inflammatory arthritis (IA), including rheumatoid arthritis (RA), psoriatic arthritis (PsA) and ankylosing spondylitis (AS), is characterized by inflammation, impaired physical function, and ultimately progressive joint damage. New pharmacological treatments have led to improved outcomes and prevention of disability over the last decades. Obviously, disease modifying anti rheumatic drugs (DMARDs) only work if taken by a patient in accordance with the prescription. According to the World Health Organization (WHO): ‘Across diseases, adherence is the most important modifiable factor that compromises treatment outcomes’. Indeed, adherence to DMARDs is still suboptimal. Non-adherence estimates between 30 and 90% [1–3], and consequently has been shown to have a negative impact on outcomes of the disease [4–6]. Adherence to treatment reflects the extent to which medication is taken as prescribed and the full benefits of DMARDs can be achieved if patients follow drug regimens [7–11]. Multiple factors have been identified that may affect medication adherence in patients with inflammatory arthritis (e.g. RA). Whereas medication beliefs, self-efficacy and the patient-clinician relationship are consistently related to medication adherence [12–16], other factors such as age, disease duration, and complexity of medication regimen show conflicting results [2, 17, 18]. Understanding which potentially modifiable factors are most important to patients and whether these factors are related to patient’s characteristics is needed to develop targeted adherence interventions [2, 18–20].

In a previous qualitative study [20], we identified facilitators and barriers to DMARDs use in patients with inflammatory arthritis using an adapted version of the Theoretical Domains Framework (TDF) [21, 22]. However, the *relative* importance of the identified facilitators and barriers of adherence for patients with inflammatory arthritis are unknown. The ranking by patients of these factors might help communication between physicians and their patients with regard to the initiation and follow up of a DMARD-treatment. Even more, a relation between the ranking of the facilitators and barriers and patient characteristics needs to be addressed; this may be useful for the development of targeted adherence interventions.

Therefore, the aim of this study is to study the ranking by arthritis patients themselves of facilitators and barriers for DMARDs adherence, using a preference scaling methodology. In addition we aim to examine whether this ranking is associated with demographic characteristics.

## Methods

### Design

This study is a cross-sectional study, ranking the importance of previously identified facilitators and barriers for adherence to DMARDs in patients suffering from inflammatory arthritis using a Maximum Difference Scaling (MaxDiff) exercise [20]. A MaxDiff exercise (or best-worst scaling) is a method that simplifies the ranking tasks for participants and provides insight into trade-offs in the decision making process (Sawtooth Software 1 (2013) The MaxDiff system/version 8 Technical Paper. Sequim: WA, Sawtooth Software. The MaxDiff method was proved to be efficacious in similar studies in rheumatology [23–25]. This study took place in 2018–2019.

### Preparatory work

For this study, facilitators and barriers for DMARD use, as identified in a previous qualitative study using focus groups and a questionnaire in patients with inflammatory rheumatic diseases was used [20]. In that study, 82 facilitators and barriers were identified, collated in the 11 domains from the adjusted Theoretical Domain Framework (TDF) [21, 22], and subsequently grouped into the higher order components Capability, Opportunity, and Motivation of the Behaviour Change Wheel (BCW) [22]. Factors were selected from these three COM components by three researchers (MV, JV, BvdB), yielding a feasible, neutrally phrased, and representative set of 35 items related to the originally identified facilitators and barriers. The 35 items were used as input for the Maximum Difference Scaling exercise. (see Table 1).

**Table 1:** Items grouped according to TDF domains and COM components

COM-B	Domain	Item
**CAPABILITY**		
1	Knowledge	• My knowledge about my arthritis medication is sufficient (e.g. how the medications work, when and how often I need to take my medications) • I understand what my treatment with medications encompasses • The reimbursement of my arthritis medications by the health insurance company • The way I have to administer my arthritis medications (injection or tablet)
2	Skills	• I can cope with my inflammatory arthritis • I have accepted my disease • I have accepted the need for my arthritis medications • I can open the medication packaging • I have good communication skills (e.g. to discuss my arthritis medications)
3	Memory and attention	• The support from family and/or friends • I can incorporate my arthritis medications into daily routines • The name and/or appearance (colour, shape) of my arthritis medications does not change • The support from my colleagues/supervisor in the workplace • The availability of aids (reminders such as electronic messages) in order to take the arthritis medications as prescribed by the physician
4	Decision-making process	• I can decide – together with my physician – about my arthritis medications • The relationship with my physician • The reimbursement of my arthritis medications by the health insurance company • I have good communication skills (e.g. to discuss my arthritis medications) • The relationship with my pharmacist
**OPPORTUNITY**		
5	Environmental context and resources	• I can get to my physician (accessibility, availability, timely access/when needed) • The reimbursement of my arthritis medications by the health insurance company • My arthritis medications are easy to use (e.g. the size of the tablet) • The name and/or appearance (colour, shape) of my arthritis medications does not change • It is easy to travel with my arthritis medication, e.g. to go abroad (able to keep my arthritis medications at a low temperature, clearance from customs/airline • My arthritis medications are expensive (a burden to society) • I can get to the pharmacy (e.g. accessibility, e.g. my arthritis medication is in stock)
6	Social influences	• The relationship with my physician • The reimbursement of my arthritis medications by the health insurance company • The support from family and/or friends • The support from my colleagues/supervisor in the workplace • The experiences of other patients with these arthritis medications • The relationship with my pharmacist
**MOTIVATION**		
7	Beliefs about capabilities	• My general health (apart from my inflammatory arthritis) • There are no side effects from my arthritis medications • I can cope with my inflammatory arthritis • I have accepted my disease • I have accepted the need for my arthritis medications • I have another chronic condition (apart from my inflammatory condition) • I can incorporate my arthritis medications into daily routines • My arthritis medications are easy to use (e.g. the size of the tablet) • The way I have to administer my arthritis medications (injection or tablet) • The availability of aids (reminders such as electronic messages) in order to take the arthritis medications as prescribed by the physician
8	Beliefs about consequences	• Arthritis medications help to reduce my symptoms • I can maintain my independence as much as possible • I *expect* the arthritis medications will help to reduce my symptoms • There are no side effects from my arthritis medications • I have accepted my disease • I *expect* to be more able to participate in social activities • I have accepted the need for my arthritis medications
9	Emotions	• I am glad arthritis medications are available • I am anxious about how my arthritis medications affect my body • I am sad I have to take my arthritis medications throughout my life • I am angry I have to take my arthritis medications throughout my life
10	Motivation and goals	• Arthritis medications help to reduce my symptoms • I can maintain my independence as much as possible • My general health (apart from my inflammatory arthritis) • There are no side effects from my arthritis medications • The relationship with my physician • I have accepted my disease • I have accepted the need for my arthritis medications • I have another chronic condition (apart from my inflammatory condition) • I can open the medication packaging • I can incorporate my arthritis medications into daily routines • My arthritis medications are easy to use (e.g. the size of the tablet) • The way I have to administer my arthritis medications (injection or tablet) • The support from my colleagues/supervisor in the workplace • What the medication regimen for my arthritis medications includes (simple or complex drug regimen) • The relationship with my pharmacist
11	Goal conflict	• My general health (apart from my inflammatory arthritis) • The support from family and/or friends • I have another chronic condition (apart from my inflammatory condition) • I can incorporate my arthritis medications into daily routines • My arthritis medications are easy to use (e.g. the size of the tablet) • The support from my colleagues/supervisor in the workplace • The experiences of other patients with these arthritis medications • The availability of aids (reminders such as electronic messages) in order to take the arthritis medications as prescribed by the physician

Note: Some determinants were grouped by patients under more than one domain

#### Pilot testing

The maximum difference scaling exercise was pilot tested among a small group of patients and care providers (patients with inflammatory arthritis, *n* = 4; psychologists, *n* = 2; and pharmacists, *n* = 2). Based on the pilot results, small changes in the wording of the items and the instructions were made to avoid misinterpretation.

### Sample size

A standard method for sample size calculations for discrete-choice experiments is lacking [26]. Neither is there a guidelines for sample size calculations when using a maximum difference scaling method [27]. Previous MaxDiff studies reported that a minimum of 100 respondents results in a reliable assessment of preferences with the MaxDiff exercise [25]. For our study, we therefore chose to recruit a convenience sample of at least 100 consecutive patients with IA from one clinic.

### Patients and measures

Patients were recruited from the rheumatology clinic (Arthritis Centre Twente, Medisch Spectrum Twente, Enschede in the Netherlands). Inclusion criteria were: 18 years or older; a diagnosis of inflammatory arthritis; using one or more DMARDs; the ability to communicate in Dutch; and informed consent. Patients with a planned visit to their rheumatologist at the hospital, were informed about this study by their rheumatologist or rheumatology nurse. If they showed interest and fulfilled the inclusion criteria, they received a letter explaining the study in more detail. The letter comprised an internet link that led them to the web-based Maximum Difference Scaling exercise, which they could complete at home. This study protocol (K17–08) was presented to the Medical Ethical Committee of the Medisch Spectrum Twente Hospital in Enschede, the Netherlands. Formal ethical approval for this study is not required under Dutch Law. Still, in accordance with the Personal Data Protection Act, informed consent was sought before the start of the Maximum Difference Scaling exercise. Taking into account an expected response rate of 20–30% [28], invitation letters were sent by mail to 450 outpatients with inflammatory arthritis using one or more DMARDs.

### Online survey

The Sawtooth software’s Lighthouse Studio MaxDiff 500 SSI Web version 8 was used to develop an online survey comprising a MaxDiff exercise (Sawtooth Software Inc. 2013) and a questionnaire to collect patient characteristics. In the MaxDiff exercise participants are shown several subsets of the possible items pertaining to medication adherence and are asked to indicate the most and least important item in each subset. In this study, each subset contained four items related to adherence.

Part 1 of the online survey assessed the following patient characteristics: sex, age, diagnosis, level of education, disease duration, and DMARDs use (which type of DMARD).

**Fig. 1:**
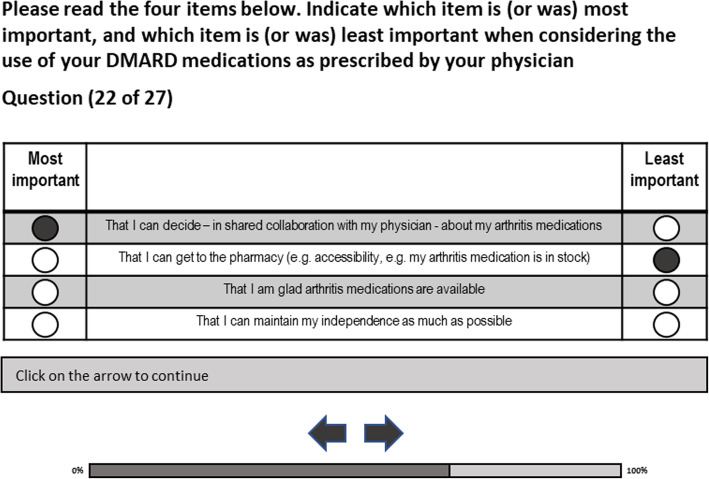
Example of a MaxDiff question

Part 2 of the online survey comprised the choice task using the maximum difference scaling method: 27 subsets were presented. Each subset contained four items related to DMARD use (Fig. 1). This approach simplifies the ranking tasks for participants, enables discrimination between ratings of different items involved in complex decisions and is not influenced by scale related biases [29]. Sawtooth software creates an optimal design of subsets based on 20,000 iterations of the exercise to ensure variation in the combination of items. An open link was created to be disseminated to the patients.

Each of the subsets had a different order of various items, to ensure that every participant would rank the item three times (27 × 4/35 = 3) and to avoid that higher importance was given to the first mentioned item. Participants were asked to choose the most important and the least important item, by answering the question: “In order to take my arthritis medication as prescribed by my physician, which item is/was most/least important to me?”

### Statistical analysis

Descriptive analyses were used to describe patient characteristics. Where appropriate, mean and standard deviation (normally distributed continuous variables), median and interquartile range (25th–75th percentile; not normally distributed continuous variables), or percentages (binary variables) were calculated. Level of education was categorized according to the International Standard Classification of Education (ISCED). To enhance international comparability, three broad groups of educational categories were constructed: no or pre-primary education, primary education and lower secondary education (ISCED 0–2); upper secondary education (ISCED 3–4); and tertiary education (ISCED 5–6). Age was grouped into tertiles (0–58/59–68/69–84 years old). The rank order and the relative importance of each item were generated by the Sawtooth software’s Lighthouse Studio, using hierarchical Bayes modelling. To facilitate interpretation, the scores were rescaled to a Rescaled Probability Score (RPS) on a scale from 0 to 100: the higher the score, the more important the item. (Sawtooth Software 1 (2013) The MaxDiff System/version 8 Technical Paper. Sequim: WA, Sawtooth Software, 2013). The rescaled probability scores added up to a value of 100, which reflects the relative importance of the item. Patients that gave inconsistent answers on the maximum difference scaling exercise (Root Likelihood below the recommended cut-off of 0.336) were excluded from the analysis. To explore a possible association between the ranking of the RPS scores and patients’ characteristics (i.e., sex, age, educational level), Kendall’s coefficient of concordance (Kendall’s W) was computed [30]. Kendall’s W, a non-parametric statistic, is used to determine the degree of agreement between groups when working with ranked data at an ordinal level of measurement. It is a normalization of the statistic of the Friedman test, and can be used for assessing agreement among raters. *Kendall’s W* ranges from 0 (no agreement) to 1 (complete agreement). A *p*-value < 0.05 allows to reject the null hypothesis that there is no agreement in ranking between groups. SPSS version 24.0 was used.

## Results

A total of 128 patients took part in the maximum difference exercise (response rate 28%). Three patients had a RLH below 0.336 and were therefore excluded from the analyses. Table 2 shows the characteristics of the participants. Most participating patients were women (60%) and most participating patients had rheumatoid arthritis (89%). The median disease duration was 15 years, IQR (7, 23). These patient characteristics were not significantly different from the former study in which the items were identified [20].

**Table 2:** Sample characteristics

	IA Patients (***N*** = 125)
*Age*, years (mean, SD)	62.7 (±12)
*Sex*	
Female	75 (60%)
*Diagnosis*	
Rheumatoid arthritis	111 (88.8%)
Ankylosing Spondylitis	4 (3.2%)
Psoriatic Arthritis	7 (5.6%)
Other diagnoses	3 (2.4%)
*Level of education*	
No or pre-primary education	38 (30.6%)
Primary education and lower secondary education	42 (33.9%)
Upper secondary education and tertiary education	44 (35.5%)
*Disease duration,* years (median, IQR)	15 (7, 23)
*Pharmacotherapy*	
Methotrexate (subcutaneous)	22 (17.6%)
Methotrexate (oral)	64 (51.2%)
Leflunomide	3 (2.4%)
Hydroxychloroquine	29 (23.2%)
Sulfasalazine	13 (10.4%)
Gold (oral)	1 (0.8%)
Infliximab	2 (1.6%)
Etanercept	15 (12%)
Adalimumab	7 (5.6%)
Rituximab	6 (4.8%)
Abatacept	5 (4%)
Tocilizumab	11 (8.8%)

*IA* Inflammatory arthritis; *SD* Standard deviation

In Table 3, the 35 items with their relative probability score are displayed and grouped according the COM-B model. The five highest ranked items were: “Arthritis medications help to reduce my symptoms” (RPS = 7.30, CI 7.17–7.44), “I can maintain my independence as much as possible” (RPS = 6.76, CI 6.54–6.97) and “I can decide –together with my physician- about my arthritis medications” (RPS = 6.48, CI 6.24–6.72), “My general health (apart from IA)” (RPS = 6.42, CI 6.08–6.76), and “I *expect* the arthritis medications will help to reduce my symptoms” (RPS = 6.32, CI 6.10–6.55).

**Table 3:** Ranking of items based on Rescaled Probability Score (RPS)

Ranking	Description	COM component (numbers: see Table 1)	RPS	CI lower	CI upper
1	Arthritis medications help to reduce my symptoms	Motivation: 8/10	7.30	7.17	7.44
2	I can maintain my independence as much as possible	Motivation: 8/10	6.76	6.54	6.97
3	I can decide – together with my physician - about my arthritis medications	Capability: 4	6.48	6.24	6.72
4	My general health (apart from my inflammatory arthritis)	Motivation: 7/10/11	6.42	6.08	6.76
5	I expect the arthritis medications will help to reduce my symptoms	Motivation: 8	6.32	6.10	6.55
6	I am glad arthritis medications are available	Motivation: 9	6.16	5.93	6.40
7	There are no side effects from my arthritis medications	Motivation: 7/8/10	5.46	5.11	5.82
8	I can cope with my inflammatory arthritis	Capability and Motivation: 7/2	5.43	5.11	5.76
9	The relationship with my physician	Capability, Opportunity and Motivation: 4/6/10	5.38	5.10	5.66
10	I can get to my physician (accessibility, availability, timely access/when needed)	Opportunity: 5	4.87	4.54	5.19
11	My knowledge about my arthritis medications is sufficient (e.g. how the medications work, when and how often I need to take my medications)	Capability: 1	4.16	3.87	4.46
12	I have accepted my disease	Capability and Motivation: 7/8/10/2	3.99	3.60	4.38
13	I understand what my treatment with medications encompasses	Capability: 1	3.79	3.49	4.09
14	I am anxious about how my arthritis medications affect my body	Motivation: 9	3.00	2.59	3.40
15	I expect to be more able to participate in social activities	Motivation: 8	2.90	2.59	3.22
16	The reimbursement of my arthritis medications by the health insurance company	Capability and Opportunity: 1/4/6/5	2.75	2.30	3.20
17	I have accepted the need for my arthritis medications	Capability and Motivation: 7/8/10/2	2.41	2.14	2.67
18	The support from family and/or friends	Capability, Opportunity and Motivation: 3/6/11	2.37	2.03	2.72
19	I have another chronic condition (apart from my inflammatory condition)	Motivation: 7/10/11	1.74	1.43	2.05
20	I can open the medication packaging	Capability and Motivation: 10/2	1.63	1.36	1.91
21	I can incorporate my arthritis medications into daily routines	Capability and Motivation: 3/7/10/11	1.45	1.24	1.66
22	My arthritis medications are easy to use (e.g. the size of the tablets)	Capability, Opportunity and Motivation:7/10/11/5	1.15	0.92	1.38
23	The name and/or appearance (color, shape) of my arthritis medications does not change	Capability and Opportunity 3/5	0.97	0.64	1.31
24	It is easy to travel with my arthritis medications, e.g. go abroad (able to keep my arthritis medications at a low temperature, clearance from customs/airline)	Opportunity: 5	0.93	0.67	1.19
25	My arthritis medications are expensive (a burden to society)	Opportunity: 5	0.90	0.67	1.12
26	I can get to the pharmacy (e.g. accessibility, e.g. my arthritis medication is in stock)	Opportunity: 5	0.84	0.69	1.00
27	I have good communication skills (e.g. to discuss my arthritis medications)	Capability: 4/2	0.82	0.69	0.95
28	The way I have to administer my arthritis medications (injection or tablet)	Capability and Motivation: 1/7/10	0.76	0.61	0.90
29	The support from my colleagues/supervisor in the workplace	Capability, Opportunity and Motivation: 6/10/11	0.63	0.47	0.79
30	The experiences of other patients with these arthritis medications	Capability, Opportunity and Motivation: 6/11	0.62	0.47	0.76
31	What the medication regimen for my arthritis medications includes (simple or complex drug regimen)	Motivation: 10	0.55	0.46	0.64
32	I am sad I have to take my arthritis medications throughout my life	Motivation: 9	0.34	0.24	0.45
33	I am angry that I have to take my arthritis medications throughout my life	Motivation: 9	0.26	0.18	0.33
34	The relationship with my pharmacist	Capability, Opportunity and Motivation: 4/6/10	0.24	0.15	0.33
35	The availability of aids (reminders such as electronic messages) in order to take the arthritis medications as prescribed by the physician	Capability and Motivation: 3/7/11	0.23	0.17	0.29

*RPS* Rescaled Probability Score; *CI* Confidence Interval

The five least important items were: “What the medication regimen for my arthritis medications includes (simple or complex drug regimen)” (RPS = 0.55, CI 0.48–0.64), “I am sad I have to take my arthritis medications throughout my life” (RPS = 0.34, CI 0.24–0.45), “I am angry that I have to take my arthritis medications throughout my life” (RPS = 0.26, CI 0.18–0.33), “The relationship with my pharmacist” (RPS = 0.24, CI 0.15–0.33), “The availability of aids (reminders such as electronic messages) in order to take the arthritis medications as prescribed by the physician” (RPS = 0.23, CI 0.17–0.30).

### Agreement in ranking between patient subgroups

Kendall’s W analyses showed that all subgroups ranked the items at a high level of agreement (Kendall’s W ranged between .97 to .99 for sex (*p* = .000), age (p = .000) and level of education (*p* = .001)) indicating that no significant difference based on sex, age or level of education, in ranking the relative importance of items important to adherence were found. *Kendall’s W* ranges from 0 (no agreement) to 1 (complete agreement). A *p*-value < .05 allows to reject the null hypothesis that there is no agreement in ranking between groups.

## Discussion

This study ranked the earlier identified facilitators and barriers of adherence to arthritis medication by patients. The five highest ranked items are “Arthritis medications help to reduce my symptoms”, “I can maintain my independence as much as possible”, “I can decide -together with my physician- about my arthritis medications”, “My general health (apart from IA)”, and “I expect the arthritis medications will help to reduce my symptoms”. All these factors are related to reducing symptoms, maintaining independency and shared decision making. The five least important ranked items were: “What the medication regimen for my arthritis medications includes (simple or complex drug regimen)”, “I am sad I have to take my arthritis medications throughout my life”, “I am angry that I have to take my arthritis medications throughout my life”, “The relationship with my pharmacist”, “The availability of aids (reminders such as electronic messages) in order to take the arthritis medications as prescribed by the physician”, suggesting practical issues are least important for arthritis patients in order to adhere to medication in this study. Categorising patients’ top five items according to the COM-B model reveals that four of the five highest ranked items are related to patient’s *motivation*, whereas one determinant is related to patient’s *capabilities*.

Our results are in line with a recently published qualitative focus group study using nominal group technique [31]. Although this latter study had an inductive approach and was consequently not based on a theoretical model, several findings were comparable. Items like the importance of patient-physician interaction and efficacy of medication were ranked high. Our study also indicated the absence of a relation between ranking of factors pertaining to adherence and demographics: sex, age and education.

Our study also confirmed that patients prefer to discuss and decide the choice of medications in close collaboration with their physician [32–34]. Patients are also more likely to adhere to a treatment that matches their preferences [35]. Tailoring treatment to patients’ medication preferences shows to be a promising strategy to improve adherence [28]. Therefore, providing the patient with unambiguous, high quality information [36] creates optimal circumstances for adherence to medication. Although practical issues such as the availability of aids to facilitate adherence were in this study ranked relatively low, such aids (e.g., application of electronic messages or reminders) can have a significant effect on adherence [37].

### Strengths and limitations

This study is the first patient oriented and theory based study ranking facilitators and barriers for adherence that can help for the development of targeted interventions to improve adherence. These findings are helpful information for healthcare professionals to guide the conversation on adherence with their patients. The mentioned factors influencing adherent behaviour can be used as a checklist for both the health professional and the patient to discuss potential adherence problems when needed. The factors ranked as most important were related to patients’ motivation, indicating that motivational support, such as motivational interviewing, could be useful to enhance adherence [38]. Furthermore, the findings of this study can be used for the development of future adherence interventions. There are some limitations in this study. The first limitation is that a selection bias in the recruitment process could have occurred. One may assume that more motivated patients, and more adherent patients were inclined to participate in this study. However, our study sample was representative for patients with inflammatory arthritis using DMARDs in the Netherlands. The second limitation is that all items related to facilitators and barriers needed to be rephrased. Obviously, rephrasing was necessary to reduce bias by using neutral wordings taking away any positive or negative valence of each facilitators and barriers [39, 40]. Most patients included in this mono centre study had rheumatoid arthritis, used conventional synthetic DMARDs (csDMARDs) or biological DMARDs (bDMARDS), and had a long disease duration, limiting the generalization of our findings. Furthermore, patients with different levels of disease activity, with different routes of administration or with comorbidities may also rank these items differently.

To examine whether the outcome of the ranking process differs in subgroup of patients (e.g., other rheumatic conditions, other DMARDs such as targeted synthetic DMARDs (tsDMARDs), patients with short disease duration, patients with different levels of disease activity) or in other countries (e.g., with different healthcare systems and insurances), future research is warranted.

### Conclusions

Our study showed the ranking of adherence-related factors by patients with inflammatory arthritis. Reducing symptoms, maintaining independency and shared decision making are considered most important for DMARDs adherence by patients. This finding can be of value to enhance communication between healthcare professionals and patients. When health professionals give attention to these items in their conversation with patients, adherence might be enhanced. Furthermore, the results are useful information for the development of interventions to optimize DMARDs adherence in IA patients. Since most highly ranked items are related to motivation according to the COM-B model, it might be useful to focus on the development of adherence interventions that capture motivational components.

## Data Availability

Due to the nature of the study, any information that could identify participants is not available. Further information is available by application to the corresponding author.

## Abbreviations

DMARDs: Disease-modifying anti-rheumatic drugs
TDF: Theoretical domains framework
IA: Inflammatory arthritis
RA: Rheumatoid arthritis
TDF: Theoretical domains framework
MaxDiff: Maximum difference scaling exercise
RPS: Rescaled probability score
BCW: Behaviour change wheel
COM-B model: Capability, opportunity and motivation-behaviour model (based on the behaviour change wheel)
Kendall’s W: *Kendall’s* coefficient of concordance
SPSS: Statistical package for the social sciences
RLH: Root likelihood
SD: Standard deviation
CI: Confidence interval
